# In vivo evaluation of a recombinant N-acylhomoserine lactonase formulated in a hydrogel using a murine model infected with MDR *Pseudomonas aeruginosa* clinical isolate, CCASUP2

**DOI:** 10.1186/s13568-021-01269-7

**Published:** 2021-07-27

**Authors:** Masarra M. Sakr, Walid F. Elkhatib, Khaled M. Aboshanab, Eman M. Mantawy, Mahmoud A. Yassien, Nadia A. Hassouna

**Affiliations:** 1 Department of Microbiology and Immunology, Faculty of Pharmacy, Ain Shams University, African Union Organization St. Abbassia, Cairo, 11566, Egypt; 2 Department of Microbiology & Immunology, Faculty of Pharmacy, Galala University, New Galala city, Suez, Egypt; 3 Department of Pharmacology and Toxicology, Faculty of Pharmacy, Ain Shams University, African Union Organization St. Abbassia, Cairo, 11566, Egypt

**Keywords:** Quorum quenching, N-acylhomoserine lactonase, *P. aeruginosa*, Burn infection

## Abstract

Failure in the treatment of *P. aeruginosa,* due to its broad spectrum of resistance, has been associated with increased patient mortality. One alternative approach for infection control is quorum quenching which was found to decrease virulence of such pathogen. In this study, the efficiency of a recombinant Ahl-1 lactonase formulated as a hydrogel was investigated to control the infection of multidrug resistant (MDR) *P. aeruginosa* infected burn using a murine model. The recombinant N-acylhomoserine lactonase (Ahl-1) was formulated as a hydrogel. To test its ability to control the infection of MDR *P. aeruginosa*, a thermal injury model was used. Survival rate, and systemic spread of the infection were evaluated. Histopathological examination of the animal dorsal skin was also done for monitoring the healing and cellular changes at the site of infection. Survival rate in the treated group was 100% relative to 40% in the control group. A decrease of up to 3 logs of bacterial count in the blood samples of the treated animals relative to the control group and a decrease of up to 4 logs and 2.3 logs of bacteria in lung and liver samples, respectively were observed. Histopathological examination revealed more enhanced healing process in the treated group. Accordingly, by promoting healing of infected MDR *P. aeruginosa* burn and by reducing systemic spread of the infection as well as decreasing mortality rate, Ahl-1 hydrogel application is a promising strategy that can be used to combat and control *P. aeruginosa* burn infections.

## Introduction

Infected burns represent one of the most dangerous causes of sepsis and death. Bacterial infections have- for long- been a common cause of mortality in burn patients (D’Avignon et al. 2010; Chen et al. 2020). Displaying increased resistance to antimicrobial agents, *P. aeruginosa* burn infections are particularly severe and have for long been of great concern. A previous study mentioned that *P. aeruginosa* caused more fatal septicaemia secondary to burn infections than the combined deaths from all other Gram negative bacteria (Holder et al. 1990). Another study also reported that *P. aeruginosa* remains a serious cause of infection and septic mortality in burn patients, particularly when nosocomially acquired (Tredget et al. 2004). A retrospective review on autopsy reports from patients with severe thermal burns reported *P. aeruginosa* among the main pathogens associated with mortality (D’Avignon et al. 2010). Another study on nosocomial infections and mortality rate of burn patients also mentioned that *P. aeruginosa* was the most frequent causative agent of burn infections (Alp et al. 2012).

One of the challenges in combating these pathogenic bacteria is due to its possession of multiple pathways for establishment of resistance against antimicrobials. The low permeability of its membrane and the constitutive expression of various efflux pumps render *P. aeruginosa* intrinsically resistant to several antibiotics. In addition to this, *P. aeruginosa* produces antibiotic-inactivating enzymes which can develop or acquire new mechanisms of resistance to antibiotics (Mesaros et al. 2007; Impey et al. 2020). All this caused *P. aeruginosa* to be among the so-termed “Superbugs” (Breidenstein et al. 2011). Alternative approaches for controlling *P. aeruginosa* burn infections are therefore of urgent need.

Interference with quorum sensing of such pathogenic bacteria represents an alternative approach for infection control. Regulation of different cell functions by quorum sensing and contribution of quorum sensing to the virulence of *P. aeruginosa* in infected burns have previously been reported (Turkina and Vikstrom 2019). Accordingly, the result of interference with quorum sensing is a reduction in virulence of this bacteria. One of efficient quorum quenchers is the lactonase enzyme present in different bacterial species including *Bacillus* (Czajkowski and Jafra 2009). AHL-lactonases catalyse lactone ring opening in the signal molecule (See-Too et al. 2018). By degrading signal molecules, namely Acyl homoserine lactone molecules, lactonase prevents accumulation of this important signal and consequently interferes with gene expression in pathogenic *P. aeruginosa.* Due to their potential importance, some previous in vivo studies have tested the ability of lactonase enzyme to control *P. aeruginosa* infection through quorum quenching. One of these studies reported that inhaled lactonase can reduce quorum sensing controlled traits in rat pneumonia (Hraiech et al. 2014). Another study tested the use of lactonase in combination with ciprofloxacin to control *P. aeruginosa* burn infection (Gupta et al. 2015). In a previous study conducted in our lab, recombinant Ahl-1 lactonase successfully reduced the production of virulence determinants in multidrug-resistant *P. aeruginosa* isolates (Sakr et al. 2018). Therefore, the aim of the present study was to evaluate the ability of a hydrogel formulation containing this recombinant lactonase, Ahl-1, to abrogate the infection of *P. aeruginosa* in a burned mouse model. To simulate complicated burn infections of *P. aeruginosa*, a MDR clinical isolate of *P. aeruginosa* was used.

## Materials and methods

### Chemicals and media

Mueller Hinton and cetrimide agar were obtained from LabM,, England. IPTG was purchased from Sigma-Aldrich Co., USA and Carboxymethyl cellulose (CMC) was purchased from El Gomhouria Co., Egypt.

### The antimicrobial susceptibility of the *P. aeruginosa* clinical isolate CCASUP2

A MDR *P. aeruginosa* clinical isolate coded CCASUP2 from Culture Collection Ain Shams University (CCASU) was isolated in our lab from a patient admitted to Ain Shams hospital. The Kirby-Bauer disk diffusion and the minimum inhibitory concentration (MIC) by microbroth dilution method were used to determine the antimicrobial susceptibility of the *P. aeruginosa* clinical isolate, CCASUP2. They were carried out as recommended by the Clinical and Laboratory Standards Institute (CLSI) guidelines (CLSI M100-S27, 2017; http://file.qums.ac.ir/repository/mmrc/clsi%202017.pdf). The Kirby-Bauer disk diffusion was done as follows: Inoculum preparation was first done by suspending freshly (18 to 24 h incubation period) isolated colonies of the test isolate, grown on Mueller Hinton agar, in isotonic saline. Turbidity was adjusted to match 0.5 McFarland standard suspension. After this inoculation of Mueller Hinton agar plates was done. A dry Mueller–Hinton agar plate was inoculated by streaking the swab over the entire agar surface. Application of the antimicrobial disks to inoculated plates then followed. The disks containing the antimicrobial agents were transferred to the surface of the inoculated plate using a sterile forceps and gently pressed. The antimicrobial disks used, their concentrations and sources are listed in Table 1. The plates were inverted and incubated at 37 °C for 16 to 18 h. Interpretation of the results was done by referring to the CLSI standard table. The susceptibilities of the isolates were recorded as susceptible (S), intermediate (I) or resistant (R) to the tested antimicrobial agents.

**Table 1:** Antimicrobial sensitivity discs, their concentrations, and sources

Antimicrobial agent	Amount per disc (µg)	Source
Co-amoxiclav (AMC)	20/10	Bioanalyse ®, Turkey
Azithromycin (AZM)	15	Oxoid®, UK
Cefotaxime (CTX)	30	Bioanalyse ®, Turkey
Ciprofloxacin (CIP)	5	
Gentamicin (CN)	10	
Meropenem (MEM)	10	Oxoid®, UK
Levofloxacin (LEV)	5	Bioanalyse ®, Turkey
Tobramycin (TOB)	10	
Aztreonam (ATM)	30	Oxoid®, UK

### Recombinant Ahl-1 lactonase

**Fig. 1:**
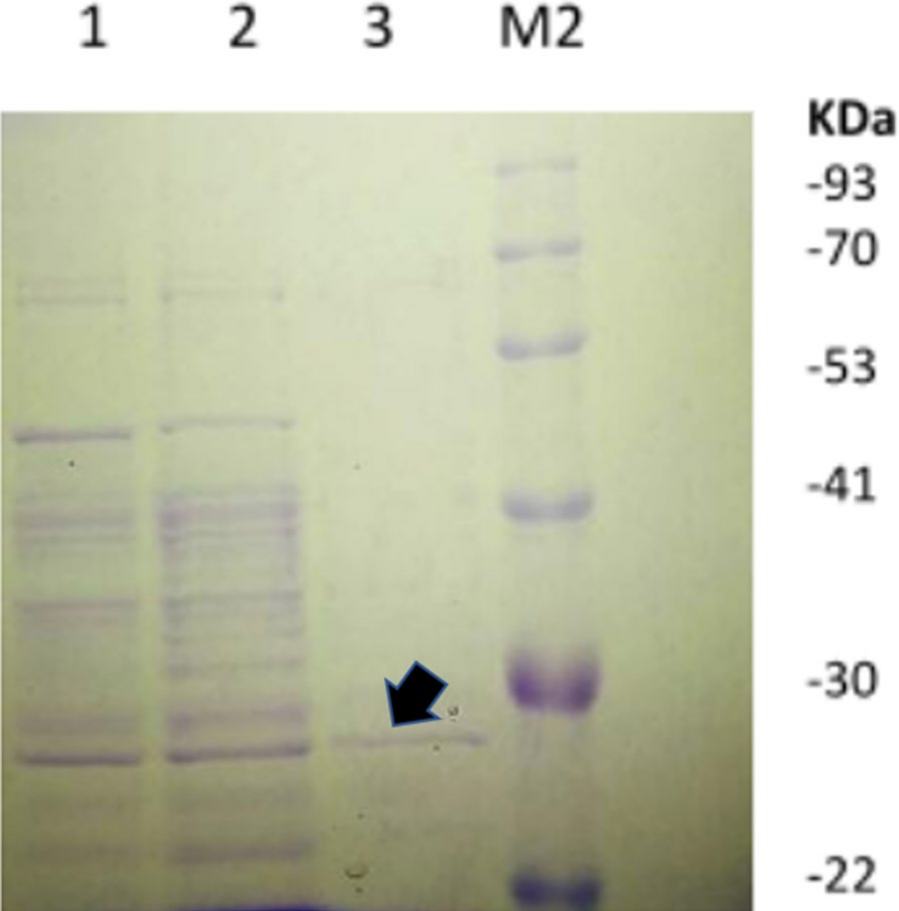
SDS-PAGE showing Ahl-1 lactonase purification using Ni–NTA spin column. Lane1: flow through, Lane 2: wash 1, Lane 3: purified Ahl-1 in the elution buffer (indicated by an arrow), M2: protein ladder (New England Biolabs, USA)

Recombinant Ahl-1 lactonase (from *ahl*-1 gene, accession code KC823046) was expressed from *E. coli* BL21 (DE3) harbouring the recombinant plasmid pET22b-*ahl*-1 (Sakr et al. 2018). Expression was performed under the control of *T7*-promotor induced by IPTG. The produced Ahl-1 lactonase was then purified using Ni–NTA spin column (Qiagen, Germany) as displayed in Fig. 1 followed by protein determination by Pierce ™ protein assay kit (ThermoFisher, Waltham, MA, USA) (Sakr et al. 2018).

### Laboratory animals

Adult Swiss albino mice, six weeks old, weighing around 25 g each were obtained from a local supplier to be used in this study. All animals were housed in open cages and the temperature was maintained at 25 °C by air-conditioning. Alternating light and dark cycles (12 h each) were applied at the animal facility of the Faculty of Pharmacy (Ain Shams University, Cairo, Egypt). Animals were allowed free access to water and antibiotic free diet (contained not less than 20% protein, 5% fiber, 3.5% fat, 6.5% ash, and a vitamin mixture). Animals were maintained in accordance with the regulations of the animal care and use committee of the Faculty of Pharmacy, Ain Shams University (ACUC-FP-ASU) which gave its consent in accordance with the National Regulations on Animal Welfare and Institutional Animal Ethical Committee. The whole study was approved by the Research Ethics Committee of the Faculty of Pharmacy, Ain Shams University, Egypt under approval no. ENREC-ASU-Nr. 62.

### Ahl-1 hydrogel preparation

This was done by mixing CMC at a concentration of 5% w/v to NPI-500 buffer (50 mM NaH2PO4, 300 mM NaCl, 500 mM imidazole, pH 8.0) containing purified Ahl-1 enzyme (drug). CMC was added portion-wise by sprinkling CMC powder with continuous mixing to guarantee the production of homogenous hydrogel. The final concentration of Ahl-1 in the gel formulation was 2 mg/ml. Another hydrogel formulation was prepared as control for the vehicle. It was prepared as 5% CMC hydrogel prepared in NPI-500 buffer (Vehicle). The prepared hydrogel had a pH of 7.7 and the spreadability coefficient (measured according to Khullar et al. 2011) was equal to 25.

## Thermal injury model

A third degree burn wound infection with a selected MDR *P. aeruginosa* was developed in mice as described previously (Dale et al. 2004). Mice were grouped into 5 groups (5 mice each). Hair was shaved from the backs of anesthetized mice by a clipper and a razor. Mice were anesthetized by exposing them to diethyl ether fumes for 10–15 s. Apart from the control group (N), a third-degree burn was induced in the backs of mice followed by establishment of *P. aeruginosa* infection in all burned mice except for mice in group (B: burned uninfected). To induce burns in the backs, a circular metal bar (10 mm in diameter and 1 mm thickness) kept on a hot plate at 95 °C for 15 s was placed on the shaved back of the mice for 20 s. immediately after the burn, all mice were injected intraperitoneally with 0.5 ml of sterile physiological saline for fluid replacement to prevent shock and 0.5 ml acetaminophen (0.25 mg/ml) was also given as a post burn analgesic. After a waiting period of 30 min, 100 µl aliquot of *P. aeruginosa* suspension (containing about 10^7^ cfu) was then applied topically to the wound area.

### Treatment

Of the three burned infected groups of mice, 2 groups received treatment as follows: Group 1: treated with Drug Group 2: treated with vehicle while the third group was left untreated (Group C). The first application of treatment was at 2 h post-infection. A weight of about 1 g hydrogel was applied topically on the infected burn. Treatment was repeated twice daily (in the morning and the evening, with a 6 h interval). With each treatment, the mice received 0.5 ml sterile saline intraperitoneally. This was repeated for three days post-infection. At 3 days post-infection, the number of animals which survived the infection was recorded. Dead animals were excluded from the groups and were only considered in calculating the mortality rates. Blood was aseptically withdrawn from the surviving animals before they were sacrificed. Animals were then sacrificed, and the livers and lungs were aseptically harvested and assayed for bacterial counts as described below. Immediately after the animals were sacrificed, dorsal skin at the wound site was also taken from the skin and subcutaneous tissue with underlying skeletal muscle for histopathological examination.

### Histopathology

Dorsal skin fixed in 10% neutral-buffered formalin (Werner et al. 2000) was used in histopathological examination. Histopathological sections were prepared from formalin-fixed, paraffin embedded tissues stained using routine procedures with haematoxylin and eosin stain. Tissues were evaluated for histopathological changes at the histopathology laboratory (Brightslide, Dr. Mohamed Abdelrazik lab, Cairo, Egypt). Analysis was performed in a blind fashion.

### Viable bacterial count

Aseptically removed lungs and livers were weighed and homogenized by using a hand-held tissue grinder in PBS. Ten-fold serial dilution of homogenized organs in PBS were prepared and plated onto cetrimide agar plates incubated at 37 °C for 24 h for quantification of bacteria. Quantification of bacteria in blood samples was also done by plating appropriate aliquots on cetrimide agar after tenfold serial dilution. Bacterial counts were calculated per gram tissue for lung and liver and per ml for blood.

### Statistical analysis

Data were analysed by a one-way ANOVA test using Graph pad Instat-3 software to determine P-values and standard deviation (Graph Pad Software Inc., USA). Results were represented as respective average values ± Standard deviation.

## Results

### Antimicrobial susceptibility profile for the *P. aeruginosa* clinical isolate, CCASUP2

Antimicrobial susceptibility analysis showed the isolate to display resistance to amoxicillin/clavulanate, cefotaxime, 2 members of fluoroquinolones (including, ciprofloxacin and levofloxacin), 2 members of aminoglycoside antibiotics (including, gentamicin and tobramycin), aztreonam and azithromycin. Results showed isolate CCASUP2 to be sensitive only to meropenem and therefore, confirmed to be a MDR isolate (Table 2).

**Table 2:** Results of antibiotic susceptibility of the clinical *P. aeruginosa* isolate, CCASUP2 against the tested antimicrobial agents

Antimicrobial agent	Susceptibility to antimicrobial agent	MIC (μg/ml)
Co-amoxiclav (AMC)	R	512
Azithromycin (AZM)	R	32
Cefotaxime (CTX)	R	64
Ciprofloxacin (CIP)	R	128
gentamicin (CN)	R	64
Meropenem (MEM)	S	2
Levofloxacin (LEV)	R	32
Tobramycin (TOB)	R	16
Aztreonam (ATM)	R	16

*S* Sensitive, *I* Intermediate resistance, *R*  resistant)

### Survival rate

Results showed that Ahl-1 hydrogel increased the survival rate of the treated animal groups and reduced mortality relative to the untreated groups, Table 3. Mortality in the group treated with Ahl-1 lactonase was zero, the same as the normal group. The (Group B), burned uninfected, showed the next higher survival rate whereas Group C (burned, infected and untreated) showed the least survival rate where only 20% survived.

**Table 3:** Percentage of animals that survived in each group

Group	Description	Animal survival percentage
Group N	Normal (control)	100
Group B	Burned uninfected (control)	60
Group C	Burned, infected, untreated (control)	20
Group 1	Burned, infected treated with Ahl-1 lactonase hydrogel (test)	100
Group 2	Burned, infected, treated with vehicle (control for vehicle)	40

### Histopathology

Histological examination of different wounded groups revealed variable degrees of tissue damage and healing process. Group C showed the most retarded wound healing process while the treated group (Group 1) demonstrated more enhanced healing process. In Group B, the group in which burn was established but left uninfected, the wound gap was found to be full of necrotic tissue, epidermal epithelial layer was lost, and inflammatory cells infiltration was observed in the dermis. As for the infected control group (Group C), coagulative necrosis along the whole skin thickness was observed in addition to a higher degree of severe inflammatory cells infiltration in dermal and subcutaneous tissue indicating the presence of heavy bacterial clusters.

**Fig. 2:**
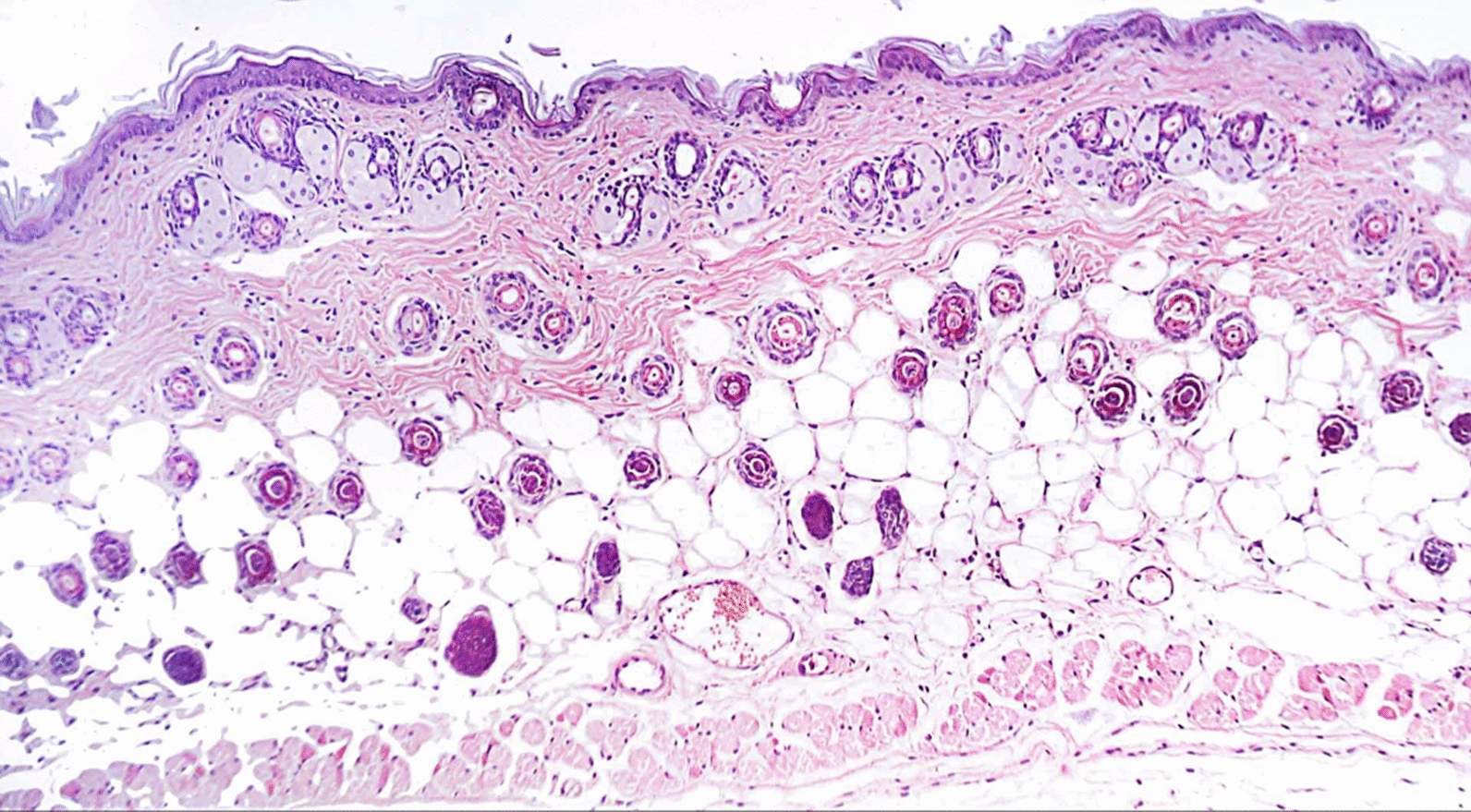
Histopathology of normal skin tissue section (Group N) showing normal intact skin layers including epidermis, dermis and subcutaneous fat and muscles (H&E 100X)

**Fig. 3:**
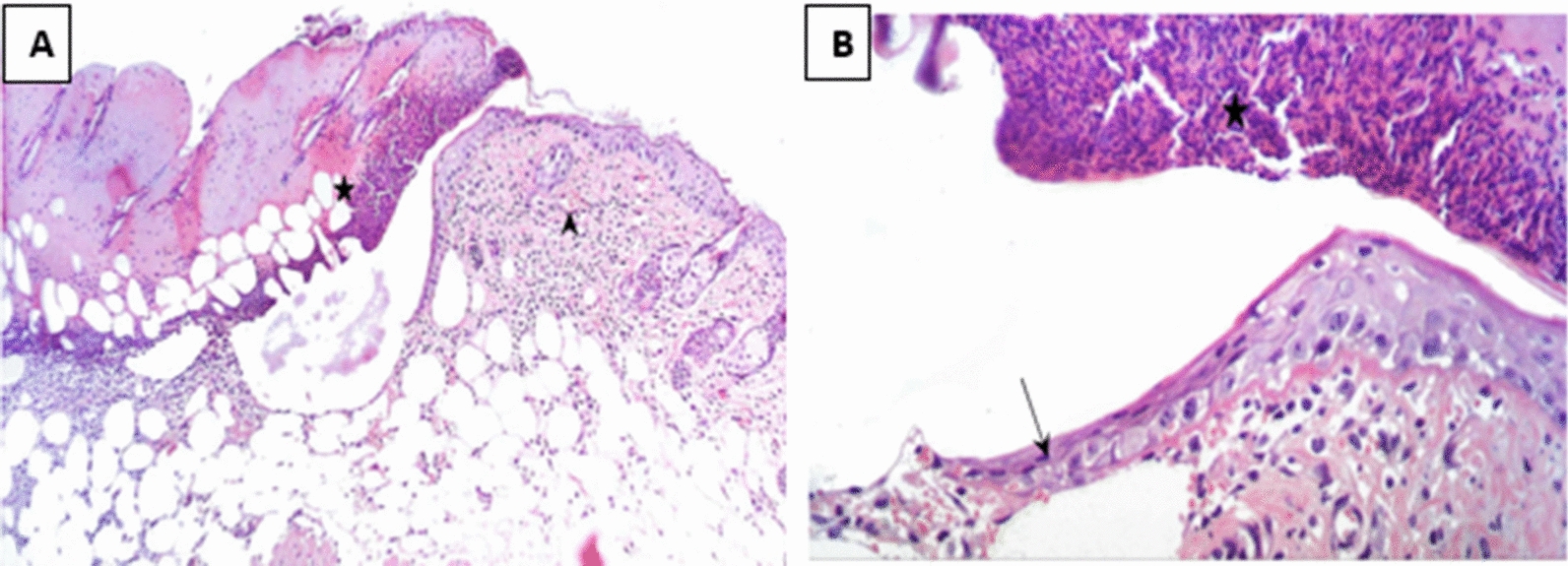
**A** Histopathology of skin from Group B (control: burned, uninfected): wound gap filled with necrotic tissue debris (star), sever inflammatory cells infiltration in dermis with scattered congested blood capillaries (arrow head). **B** Higher magnification from (**A**) showing wound epithelium edge (arrow) covered by necrotic tissue (star) and underlying damaged dermal layer (H&E 400X)

**Fig. 4:**
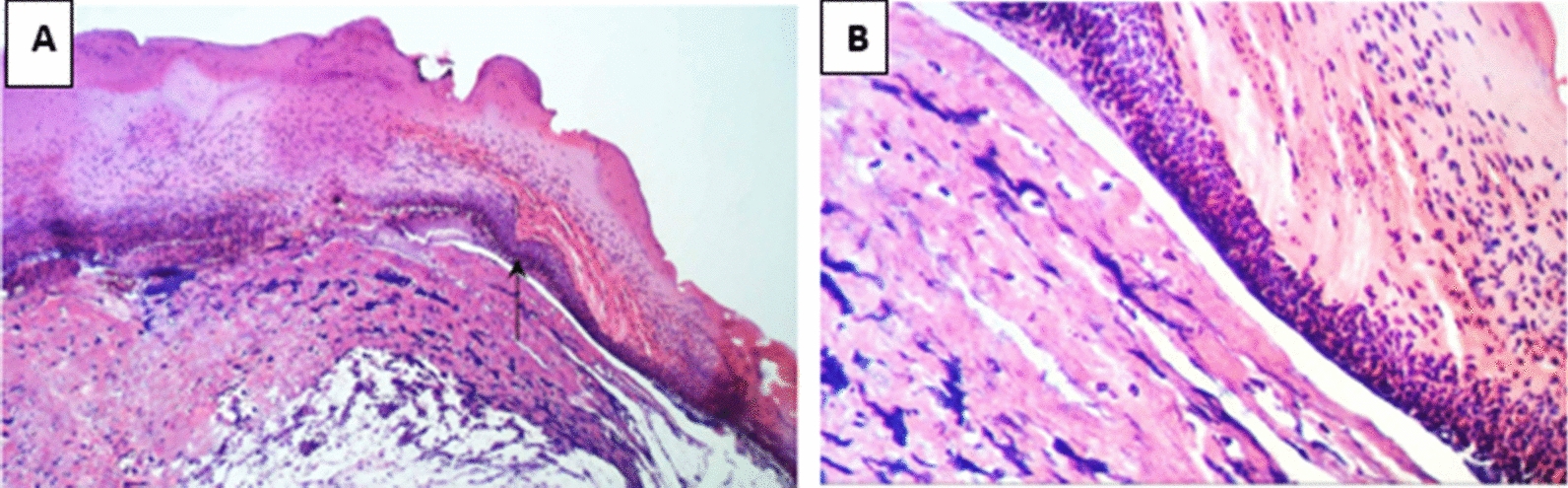
**A** Histopathology of skin from Group C (control: burned, infected, untreated). showing wounded area with coagulative necrosis along the whole skin thickness associated with sever inflammatory cells infiltration in dermal and subcutaneous tissue associated with heavy bacterial clusters (arrow). **B** Higher magnification from (**A**) showing epidermal necrosis with many inflammatory cells and bacterial clusters in deeper tissue (H&E 400X)

**Fig. 5:**
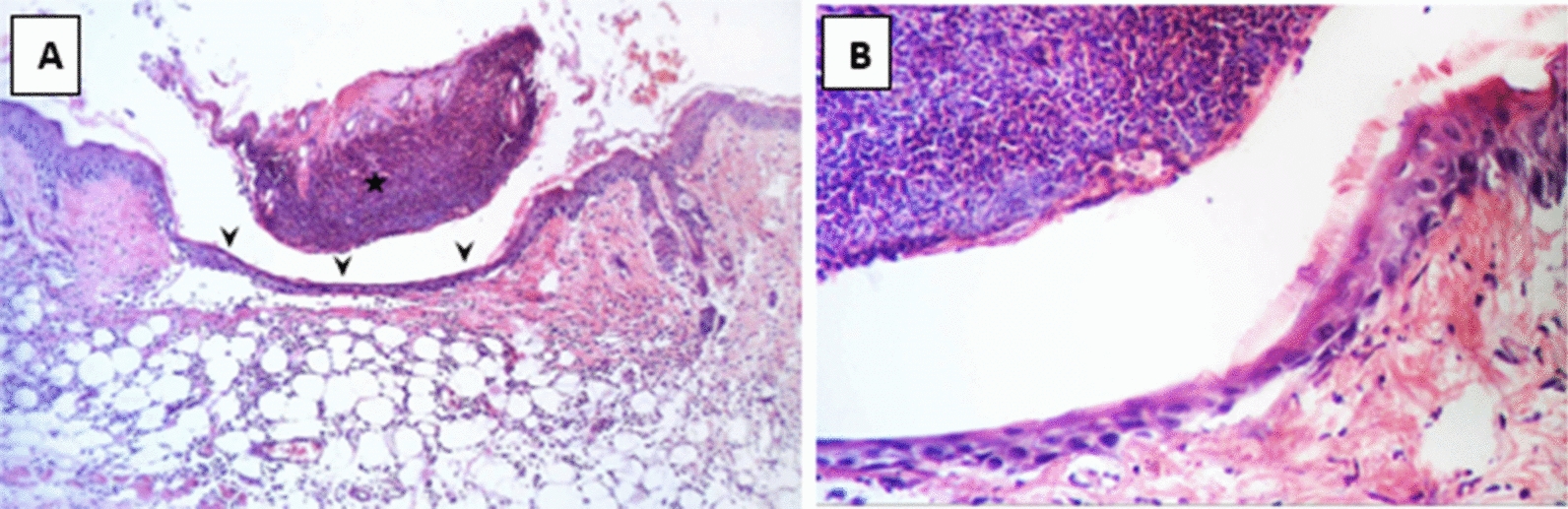
**A** Histopathology of skin from Group 1 (test: burned, infected, treated with Ahl-1 hydrogel) Showing complete epithelial cells bridging (arrow head) the wound gap under crust from necrotic tissue debris and inflammatory cells (star), inflammatory cells infiltration in dermal, subcutaneous fat layer with many activated fibroblasts. **B** Higher magnification from (**A**) showing complete epithelial cells bridging the wound gap under necrotic tissue with newly formed collagen fibres in underlying dermis (H&E 400X)

**Fig. 6:**
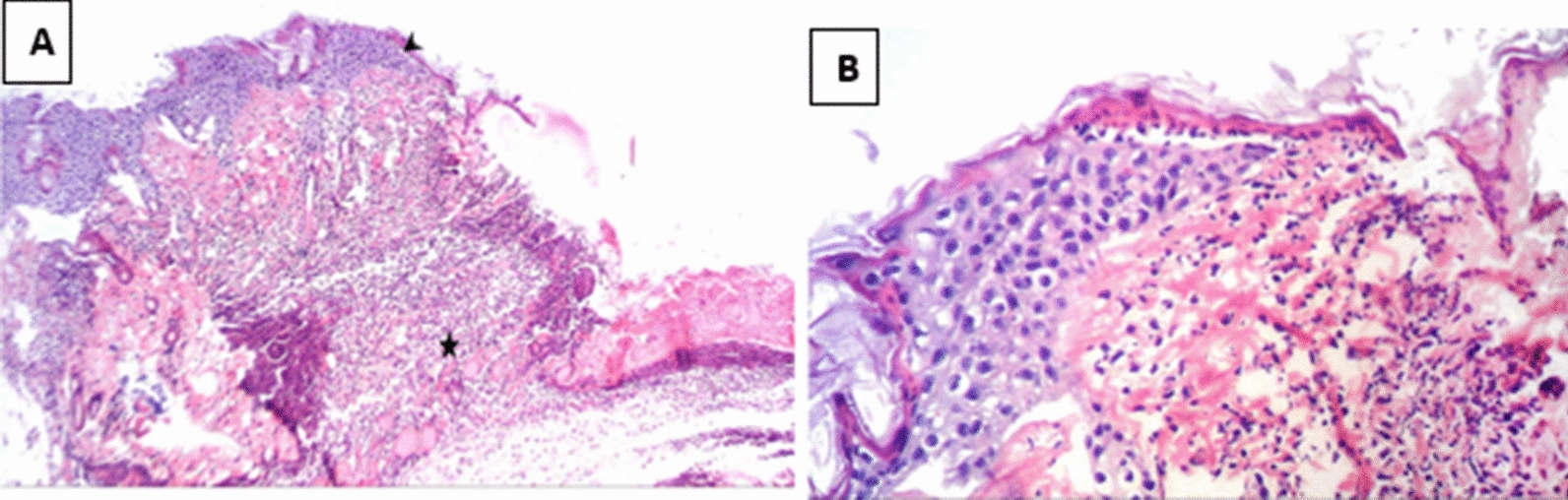
**A**. Histopathology of skin from Group 2 (burned, infected, treated with vehicle). Wounded region with incomplete epithelial bridging by epithelial edge (arrow head), attenuated dermis with area of denudation on the right side and sever inflammatory cells infiltration in dermal full thickness as well as subcutaneous tissue and underlying skeletal muscles (star). **B** Higher magnification from (**A**) showing wound epithelial edge with underlying inflammatory cells infiltration in dermal layer (H&E 400X)

In Group 2 (treated with a hydrogel formulation containing only the buffer NPI-500), the wounded region was observed to have incomplete epithelial bridging by epithelial edge, attenuated dermis with area of denudation in addition to severe inflammatory cells infiltration in dermal full thickness as well as subcutaneous tissue and underlying skeletal muscles. As for the group treated with Ahl-1 hydrogel, examination revealed the regeneration of epithelium as complete epithelial cells bridging was observed at the wound gap under crust from necrotic tissue debris and inflammatory cells. Numerous activated fibroblasts were observed in the subcutaneous fat layer as well as newly formed collagen fibres in underlying dermis. Figures 2, 3, 4, 5 and 6 show the examined skin from the different groups displaying the histopathological features observed in each of them.

### Viable bacterial count

Results showed that there was a decrease of up to three logs of bacterial count in the blood samples of animals treated with Ahl-1 hydrogel relative to the control group. The same group showed a decrease of up to 4 logs and 2.3 logs of bacteria in lung and liver samples relative to the control group (Group 2). Statistical analysis using one-way analysis of variance (ANOVA) showed significant differences in counts between the treated group and other groups. This indicates that Ahl-1 lactonase successfully reduced systemic dissemination of *P. aeruginosa* from site of infected burn. Table 4 displays the results of *P. aeruginosa* viable count in different organs of the tested animals in various groups.

**Table 4:** Log viable count of *P. aeruginosa* in different organs of animals in different animal groups (P value < 0.001)

Group	Description	Mean bacterial count ± SD		
		Blood Log no. of CFU/ml	Lung Log no. of CFU/g	Liver Log no. of CFU/g
Group N	Normal (control)	0.0 ± 0.0	0.0 ± 0.0	0.0 ± 0
Group B	Burned uninfected (control)	1.84 ± 0.39	1.69 (0.5)	1.602 ± 0.28
Group C	Burned, infected, untreated (control)	4.68 ± NA^a^	4.7 ± NA^a^	5.05 ± NA^a^
Group 1	Burned, infected treated with Ahl-1 lactonase hydrogel (test)	0.536 ± 0.09	1 ± 0.29	2.3 ± 0.41
Group 2	Burned, infected, treated with vehicle (control for vehicle)	3.78 ± 1.2	5.114 ± 0.3	4.6 ± 0.56

^*^NA Not available

## Discussion

Infected burns have always been of great concern. Acute burn wounds damage the skin barrier and suppress the immune system increasing the liability of the patient to acquire a bacterial infection (McVay et al. 2007). Despite advances in health care, Gram negative bacterial infection namely MDR *P. aeruginosa* remains among the most common causes of bacteria related mortality where colonization of a burn wound often leads to a disseminated infection, and sometimes a septic shock (D’Avignon et al. 2010). Several approaches have been attempted to control burn infection of such virulent pathogen well known for its high resistance including the use of nanosheets loaded with antibiotics (Saito et al. 2012). Due to *P. aeruginosa* well known developing resistance to antibiotics, alternative approaches including use of natural products (Hazrati et al. 2010) and phage (McVay et al. 2007) to combat *P. aeruginosa* infections in burns have been studied. However, due to some disadvantages of these approaches which include allergy associated with natural products (Helbling et al. 1992) and the drawbacks of phage therapy (Loc-Carrillo and Abedon 2011), quorum quenching approach seems a better option. Among the quorum quenching compounds that present a promising tool for infection control is lactonase enzyme. Previous studies reported the ability of lactonase enzymes produced by different bacterial species to supress the expression of virulence genes of pathogenic bacteria (Mayer et al. 2015; Sakr et al. 2018; Morohoshi et al. 2019). In addition to this, a previous study reported the non-toxicity of lactonase administrated topically towards the host and it did not induce inflammation or apoptosis in macrophages (Gupta et al. 2015). Studies that tested the stability of recombinant lactonase enzymes also showed that the enzyme was thermostable (Sakr et al. 2013; Zhang et al. 2019) and that its relative activity remained after storage at low temperatures for three months (Zhang et al. 2015). Accordingly, the present study aimed at testing the effect of recombinant Ahl-1 lactonase formulated as a hydrogel on MDR *P. aeruginosa* infected burn. Cellulose is a highly abundant biopolymer with unique properties (Kayra et al. 2018), so it was used in the hydrogel preparation. To achieve this, a clinical *P. aeruginosa* was isolated from a patient suffering from various complications and displaying non-responsiveness to various antimicrobial agents used in therapy. The Antibiogram analysis revealed a high level of resistance possessed by the isolate towards different antimicrobial agents. Such isolate, CCASUP2, represents with no doubt a threatening situation to a patient with an infected burn. To assess the ability of a recombinant lactonase Ahl-1 formulated as a hydrogel to attenuate the pathogenicity of such MDR isolate, a thermal injury model was establishment in mice and infected with MDR *P. aeruginosa* isolate CCASUP2. After establishment of the infection, the prepared hydrogel was used in the treatment of the infected burn. Survival rates of the treated group was calculated and compared to other control groups. Histopathological examination of the burn site was done as well as determination of the viable bacterial count in blood and at distant organs to assess the dissemination of infection. Results of this study revealed that Ahl-1 lactonase increased the survival rate where all the animals treated with the purified drug survived. This result comes in accordance with previous studies. A previous study reported ability of lactonase to reduce mortality in burn infection in combination with ciprofloxacin (Gupta et al. 2015). Two other related studies tested the efficacy of lactonase in controlling *P. aeruginosa* infection using mouse and rat acute pneumonia models (Migiyamaa et al. 2013; Hraiech et al. 2014). Results in these studies also reported reduction in mortality when lactonase was administrated. Results also showed that hydrogel (vehicle) without the enzyme increased survival rate compared to the untreated mice. This could probably be attributed to the moisturizing effect of the hydrogel and its acting as a physical barrier that protected the wound as reported in a previous study that mentioned the advantages of hydrogels burn in wound care (Madaghiele et al. 2014). A study in 2019 also reported the ability of lactonase to reduce virulence determinants in *P. aeruginosa* isolates collected from burned patients (López-Jácome et al. 2019). The combination of quorum sensing inhibitor and quorum sensing enzyme was reported by Fong and co-workers to supress multiple quorum sensing pathways in *P. aeruginosa* isolate (Fong et al. 2018).

Histopathological examination of the site of infected burn revealed that topical treatment with lactonase hydrogel suppressed local wound infection and induced regeneration of skin. To ensure the accuracy of results and attribute the healing activity solely to lactonase, an extra control group other than the untreated group was designed in the study. In this control group (Group 2), a hydrogel prepared using the vehicle only without lactonase was applied topically on the infected burn and the outcome was assessed. In the same manner, group B in which established burns were left uninfected was used to compare the damage caused by the *P. aeruginosa* infection in the other groups relative to this group. Ability of lactonase enzyme to control the infection of *P. aeruginosa* in burns was previously reported. A study by Gupta *et. al*. reported that the simultaneous use of topical lactonase in addition to intraperitoneal ciprofloxacin enhanced skin regeneration in a *P. aeruginosa* infected burn (Gupta et al. 2015). However, our study here has the advantage of testing the effect of lactonase alone in addition to using a MDR *P. aeruginosa* isolate in inducing the infection. Related studies in which lactonase successfully reduced virulence in an in vivo model through quorum quenching included a study in which co-injection of lactonase with *Aeromonas hydrophila* in zebra fish decreased the mortality rate and delayed the mortality of fish (Cao et al. 2012). Another recombinant lactonase was also reported to control *Erwinia carotovora* infection (Dong et al. 2000). Another study used paraoxonase enzyme which is closely related to lactonase to control *P. aeruginosa* infection in *Drosophila* (Stoltz et al. 2008). Human serum paraoxonase seems another promising quorum quenching enzyme as reported by another study (Aybey and Demirkan 2016).

In addition to promoting healing at the local area of infection, Ahl-1 successfully prevented the systemic spread of the MDR *P. aeruginosa* infection to other organs as displayed by the viable count determination. In the previous study by Gupta and coworkers (2015), topically applied lactonase reduced bacterial load in blood, lung and liver after three days of treatment by a range of 1.5 to 2.4 logs while bioburden reduction in our study here reached 4 logs of bacterial count. This indicates the higher efficacy of the recombinant produced Ahl-1 lactonase used in the present study. By supressing systemic spread of the infection to other organs, lactonase is expected to reduce mortality in patients with *P. aeruginosa* infected burns as a previous study reported that patients requiring systemic treatment secondary to a burn infection showed the highest mortality rate (21.2%) (Branski et al. 2009).

In conclusion, results of this study revealed that Ahl-1 lactonase formulated as a hydrogel for topical application has the ability to control the infection of MDR *P. aeruginosa* in burns reducing both systemic spread to other organs and mortality rate. In addition to that, it promotes healing at the injury site as depicted from the histopathological examination. Epithelium was restored and activated fibroblasts were observed. In conclusion, recombinant Ahl-1 lactonase used in this study reduces the in vivo virulence of MDR *P. aeruginosa* clinical isolates suppressing its infection in burned mouse model. It is a promising new tool against such pathogen and a weapon against its threat to burned patients. However, the obtained findings have to be confirmed through further in vivo models. Testing Ahl-1 lactonase in combination with antibiotics in addition to experimenting its use clinically is important to ensure its safety, efficacy and therefore, confirm its potential use in human.

## Data Availability

All the data is presented and available in the manuscript.
